# Supplementary material for: Formate Utilization by the Crenarchaeon Desulfurococcus amylolyticus
Source: Microorganisms. 2020 Mar 23;8(3):454. doi: 10.3390/microorganisms8030454 (PMC7143981; doi:10.3390/microorganisms8030454)
Supplement: Supplementary file 1 [file microorganisms-08-00454-s001.zip › Supplementary_Table_4.docx]

**Supplementary Table S4**: Gibbs values of expected intermediates and metabolic end products from formate metabolisation by *D. amylolyticus* at 25°C and 80°C**^*^**

| **Reaction** | **T [°C]** |  | **∆G^0'^**  **[kJ mol^-1^]** | **∆G^0'^/ formate [kJ mol^-1^]** |
| --- | --- | --- | --- | --- |
| 2Formate 🡪 Formaldehyde + CO_2(g)_ + H_2_O | 25 |  | 10.09 | 5.04 |
|  | 80 |  | 10.13 | 5.06 |
| $2CHOO^{-}+ 2H^{+} \overset{\leftrightarrow}{} {CH}_{2}O+CO_{2}+H_{2}O$ |  |  |  |  |
| Formate 🡪 CO_2(g)_ + H_2(g)_ | 25 |  | -3.53 | -3.53 |
|  | 80 |  | -10.72 | -10.72 |
| $CHOO^{-}+H^{+}\overset{\leftrightarrow}{} CO_{2}+ H_{2}$ |  |  |  |  |
| 6Formate 🡪 Ethanol + 4CO_2(g)_ + 3H_2_O  $6CHOO^{-}+ {6H}^{+} \overset{\leftrightarrow}{} C_{2}H_{5}OH+4CO_{2}+{3H}_{2}O$ | 25 |  | -125.28 | -20.88 |
|  | 80 |  | -122.85 | -20.48 |
|  |  |  |  |  |
| 4Formate 🡪 Acetate + 2CO_2(g)_ + 2H_2_O  4$CHOO^{-}+3H^{+} \overset{\leftrightarrow}{}{CH}_{3}COO^{-}+2CO_{2}+{2H}_{2}O$ | 25 |  | -109.03 | -27.26 |
|  | 80 |  | -105.32 | -26.33 |
|  |  |  |  |  |
| 10Formate 🡪 Acetate + Ethanol + 6CO_2(g)_ + 5H_2_O | 25 |  | -234.31 | -23.43 |
| $10CHOO^{-}+9H^{+} \overset{\leftrightarrow}{}{CH}_{3}COO^{-}+ C_{2}H_{5}OH+6CO_{2}+{5H}_{2}O$ | 80 |  | -228.17 | -22.82 |

^*^∆G^0^: *P*_SAT_, pH = 0. ∆G^0'^: *P*_SAT_, pH = 7
