# Supplementary figures and images for: Formate Utilization by the Crenarchaeon Desulfurococcus amylolyticus
Source: Microorganisms. 2020 Mar 23;8(3):454. doi: 10.3390/microorganisms8030454 (PMC7143981; doi:10.3390/microorganisms8030454)

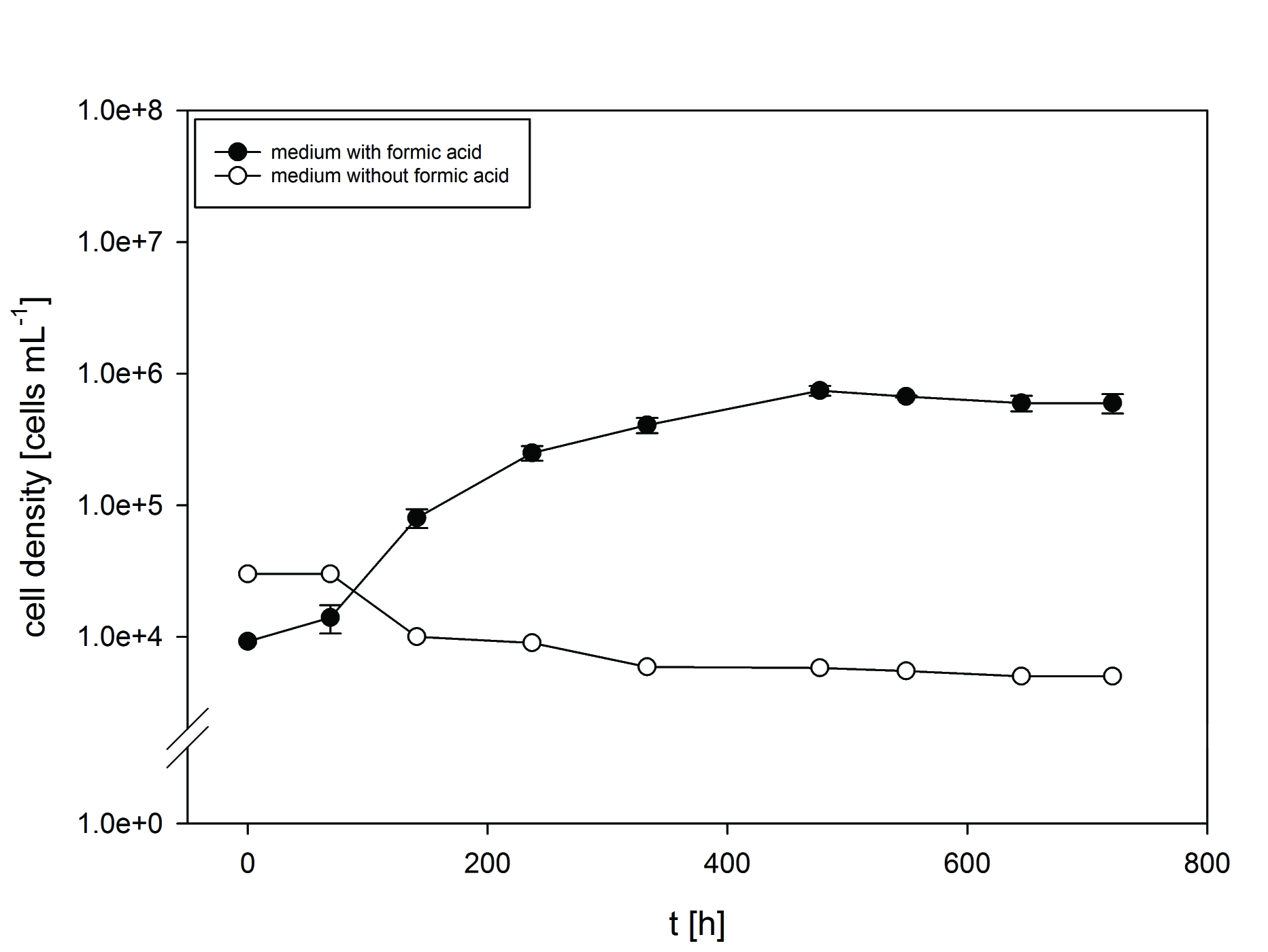

Supplement: Supplementary file 1 [file microorganisms-08-00454-s001.zip › Supplementary_Figure_1.TIF]
